# Public Health and Political Corporate Social Responsibility: Pharmaceutical Company Engagement in COVAX

**DOI:** 10.1177/00076503231158600

**Published:** 2023-04-13

**Authors:** Markus Scholz, N. Craig Smith, Maria Riegler, Anna Burton

**Affiliations:** 1 FHWien University of Applied Sciences of WKW, Austria; 2 Technische Universität Dresden, Germany; 3 INSEAD, Fontainebleau, France; 4 Austrian Institute of Economic Research (WIFO), Vienna, Austria

**Keywords:** access to medicine, COVAX, Covid-19, multistakeholder partnerships, political corporate social responsibility

## Abstract

Pharmaceutical companies developed Covid-19 vaccines in record time. However, it soon became apparent that global access to the vaccines was inequitable. Through a qualitative inquiry as the pandemic unfolded (to mid-2021), we provide an in-depth analysis of why companies engaged with the Covid-19 Vaccines Global Access Facility (COVAX), identifying the internal (to the company) and external factors that facilitated or impeded engagement. While all producers of the World Health Organization (WHO)-approved vaccines engaged with COVAX, our analysis highlights the differential levels of COVAX engagement and identifies contractual obligations, opportunities and company strategy, and reputational pressures as key explanatory factors. We discuss our empirical findings relative to the literature on political corporate social responsibility (PCSR). Accordingly, we question whether pharmaceutical companies lived up to their responsibilities as corporate citizens and conclude that they failed to fulfill the implied responsibility of combating inequitable vaccine distribution. We conclude with implications of our research for practice, in relation to the challenges of global access to Covid-19 vaccines and for access to medicines more generally.

Covid-19 triggered the biggest global health crisis since the Second World War. It brought health care systems to their knees and caused millions of deaths (The Economist, 2022; Johns Hopkins University, 2022; World Health Organization [WHO], 2020). The pharmaceutical industry had a potentially vital role in mitigating the effects of this disastrous scenario. Vaccines offered the best hope to soften the impacts of the pandemic, if not to end it altogether (Srivastava, 2021). Here, the interplay of business and society becomes visible in extraordinary clarity. Could business respond effectively to a pressing social need? Would it do so in ways that served all of humanity as it faced the onslaught of a pandemic?

Pharmaceutical companies succeeded in developing effective vaccines in under a year, a process that typically takes 5 to 15 years (Johns Hopkins University, 2021). Given doubts about the industry (Scholz & Smith, 2020), this was a big win for research-based pharma—and for society. Nonetheless, huge challenges remained in securing equitable *access* to these vaccines for much of the world. First, there was initially a massive shortage of vaccines due to global production capacity limitations as well as logistical challenges (The Economist, 2021a). Second, high-income countries were acquiring enormous quantities of vaccines, leaving only minimal volumes for the populations in low-income countries (Shah, 2020).

The Covid-19 Vaccines Global Access Facility (COVAX), a multistakeholder partnership, was created in April 2020 to provide equitable access to Covid-19 vaccines. As of mid-2021, COVAX could claim only limited success. The Director General of the WHO, explaining that COVAX was hampered in its efforts to reach its goals, called on countries *and companies* to do more (Tedros, 2021).^1^ Many claimed that inequitable access to Covid-19 vaccines constituted a major ethical problem of human rights (Bachelet, 2021). The situation highlighted significant theoretical and practical questions of business and society.

In this article, we explore the *political corporate social responsibility* (PCSR) of pharmaceutical companies to collaborate with COVAX, as the primary means by which they could contribute to providing equitable access to Covid-19 vaccines. PCSR, in the dominant form as popularized by Andreas Scherer and Guido Palazzo (cf. Scherer et al., 2006; Scherer & Palazzo, 2007, 2011), can be seen to offer a normative justification for why vaccine producers would have a responsibility to engage with COVAX and thereby help mitigate inequitable access to vaccines.

Our research contributes to the theoretical debate on PCSR by providing a more nuanced picture of why companies engage with broader business and society questions. We provide an analysis of the role and motivations of pharmaceutical companies in potentially addressing the “grand challenge” of equitable access to vaccines during a pandemic. Our phenomenon-driven study explores the engagement of Covid-19 vaccine-producing companies with COVAX between the start of the pandemic and mid-May 2021.^2^ We believe this historical snapshot is the first in-depth empirical analysis of company engagement with COVAX. The research questions we aim to answer are the following:

**Research Question 1:** Why do pharmaceutical companies engage with COVAX?
**Research Question 2:** What factors motivate, facilitate, and hinder company engagement with COVAX?

We proceed as follows. Section “Research Context” describes the research context, overviewing COVAX and pharmaceutical company engagement, and further elaborates on PCSR theory and the responsibilities of pharmaceutical companies. Section “Method” describes our research methodology. Section “A Thematic Account of Company Engagement With COVAX” reports the results of our empirical study, structured by the factors that help explain company engagement with COVAX. Section “Understanding the Differential Engagement of Companies With COVAX” discusses the reasons for differential engagement of vaccine producers with COVAX. Finally, in section “Discussion and Conclusion,” we discuss our findings relative to the extant literature on PCSR, suggesting how they both support and extend existing theory. We conclude by noting the research limitations and directions for further research.

## Research Context

### A Brief Overview of the COVAX

With the pandemic growing rapidly, the Access to Covid-19 Tools (ACT) Accelerator was launched on April 24, 2020. It brought together governments, industry, civil society, and global health organizations^3^ to accelerate the development, production, and equitable access to Covid-19 diagnostics, treatments, and vaccines (Gavi, 2021). COVAX is the vaccine pillar of the ACT Accelerator.^4^ The stated goal of COVAX was “to help end the acute phase of the global pandemic by the end of 2021 by providing access to at least 2 billion doses of safe and effective Covid-19 vaccines to the most vulnerable in all participating economies” (Gavi, 2021).

Countries signed up to COVAX to provide financing (supplemented by contributions from other parties) and obtain vaccines, with fully self-financing (richer) countries primarily funding the facility and either paying for both their own supply and that of poorer countries or, if they had secured sufficient supplies via bilateral deals, only financing the supply for (poorer) countries (The Economist, 2021a). COVAX established Advance Market Commitments (AMCs) with manufacturers to procure the vaccines, with the United Nations International Children’s Emergency Fund (UNICEF) handling the vaccine logistics. The WHO allocation principles provided for WHO-approved vaccines to be distributed initially to all countries in proportion to their population size, enabling every country to start immunizing the most vulnerable groups (WHO, 2021b). COVAX had defined three priority stages: in the first stage, all critical health and social care workers globally would be vaccinated (roughly 3% of the global population); followed by high-risk and older aged groups in Stage 2 (circa 20%); and further priority groups in Stage 3.

By mid-May 2021, 14 companies had successfully developed vaccines and applied for Emergency Use Listing (EUL) at the WHO (WHO, 2021a).^5^ Given that COVAX could only buy and distribute WHO-approved vaccines, our analysis focuses on these companies.

### The Development of Company Engagement With COVAX

Governments initiated conversations with pharmaceutical companies as early as May 2020 to secure doses for their own populations through advance purchase agreements. COVAX also engaged in conversations with vaccine producers starting in May 2020, although it did not have the necessary funding until September. After signing a manufacturing agreement with Oxford University in April 2020, AstraZeneca was the first pharmaceutical company to sign a COVAX deal. The same day, AstraZeneca also completed a licensing agreement with Serum Institute of India (SII), the world’s largest vaccine manufacturer, to increase production volumes to serve low- and lower-middle-income countries. In September, SII became the second manufacturer to commit to COVAX. The Sanofi–GlaxoSmithKline collaboration followed in October.

In November 2020, interim data from clinical trials of the Pfizer/BioNTech, Moderna, and AstraZeneca/Oxford vaccines provided promising indications of their efficacy against Covid-19. AstraZeneca and Johnson & Johnson significantly increased their commitments to COVAX in mid-December, providing COVAX with pledges for nearly two billion vaccine doses at the end of 2020—although no vaccines had yet been approved by regulatory authorities.

COVAX finally came to terms on an advance purchase agreement with Pfizer/BioNTech in late January 2021—although this was for up to 40 million doses only. Shortly after, SII agreed to COVAX options on an additional amount of 1.1 billion doses of AstraZeneca and Novavax vaccines. In addition, three Chinese vaccine-producing companies (Sinovac, Sinopharm, and CanSinoBio) applied to join the initiative in late January 2021. The Russian Direct Investment Fund (RDIF) applied in March 2021 to participate in COVAX, offering the Sputnik V vaccine.

Finally, in early May, advance purchase agreements were signed with Moderna to secure up to 500 million vaccine doses even though statements on discussions had been announced as early as October 2020.^6^

### Motivations for Company Engagement With COVAX Against the Backdrop of PCSR

Our focus in this article is on the motivations for company engagement with COVAX against the backdrop of normative PCSR theory. In brief, PCSR “suggests an extended model of governance with business firms contributing to global regulation and providing public goods” (Scherer & Palazzo, 2011, p. 901).^7^ The concept is defined against the backdrop of the shortcomings of a neoliberalist conception of democracy, in which companies are viewed as economic actors whose responsibilities are limited to complying with hard laws and contractual obligations. In this view, companies should only engage with social problems (e.g., nonequitable access to vaccines) if this engagement is beneficial not only for society but also increases their profits (Friedman, 1962, 1970). This instrumental conception of corporate social responsibility is challenged by PCSR scholars (among many others) who argue that the “clear-cut division of labour” paradigm as dominant in the neoliberalist conception of democracy is insufficient when it comes to tackling grand challenges like migration issues, climate change, or, as most relevant for this article, a global health crisis (Scherer & Palazzo, 2011, p. 922).

PCSR scholars advance a different concept of governance and democracy. Against the backdrop of globalization and the waning influence of governments to regulate in ways that adequately constrain corporate behavior and combined with the decreasing capacity of traditional state actors to address grand challenges sufficiently, PCSR proponents argue that companies should engage in public deliberation and self-regulation. Furthermore, multinational enterprises should (and do already) engage in public goods provision such as public health, education, social security, and protection of human rights to fill the gaps left by governments unwilling or unable to act (Scherer et al., 2013, 2016; Scherer & Palazzo, 2007, 2011). From the PCSR perspective, it seems reasonable to claim that vaccine-producing companies would have a political responsibility to engage to a significant extent with COVAX and foster equitable access to Covid-19 vaccines. We contend that the behavior of vaccine-producing pharmaceutical companies in their engagement with COVAX (during our investigation period) provides a critical test case for PCSR.

While PCSR is generally acknowledged as one of the dominant streams in business ethics (Scholz et al., 2019), it has also met heavy criticism. Whelan (2012, p. 717) claims that PCSR appears to contradict the dominant scholarly assumption that (Western) multinational companies are mainly motivated to generate profits. In reference to Stout (2012, p. 3), he goes on to criticize the PCSR literature because it seems to overlook the prevalent business focus placed on shareholder wealth maximization. More recently, Rhodes and Fleming (2020) have even argued that we should forget the idea of PCSR altogether as it misconstrues the motivations of capitalist firms as being nonexclusively instrumental.

This criticism notwithstanding, we concur with the general normative perspective of PCSR (Scherer et al., 2006; Scherer & Palazzo, 2007, 2011)—that companies, especially those in the pharmaceutical industry, have extended responsibilities and should engage in the provision of public goods, such as in public health and the protection of human rights.^8^

PCSR’s point about a failure of public institutions is especially relevant to the question of fair access to Covid-19 vaccines. As of mid-2021, governmental institutions had failed to adequately anticipate, prevent, or redress global inequitable access to vaccines, notwithstanding the efforts described in our timeline in the previous section. In addition, moral arguments for access to medicines generally, and Covid-19 vaccines more specifically, are certainly well grounded in human rights. First, because of the fundamental human right to health.^9^ Second, because of the restrictions on human rights that stem *directly* from government responses to the pandemic, such as constraints on freedom of assembly and access to education (Santoro & Shanklin, 2020), but also *indirectly* from the economic consequences of the pandemic. Michelle Bachelet (2021), the acting UN High Commissioner for Human Rights, stated that “the human rights impact of our global failure to vaccinate widely enough is profound. It is driving sharply divergent economic recoveries from the first waves of the pandemic.”

Thus, from a normative PCSR perspective, it can be argued that when national and global governance institutions fail to provide fair access to lifesaving vaccines, companies need to step up and help close this gap. From an access to medicine perspective, Leisinger (2009) strongly suggests that pharmaceutical companies in collaboration with the international community should ensure access to affordable, essential drugs in developing countries, offering recommendations also supported by the International Federation of Pharmaceutical Manufacturers & Associations (IFPMA).

While we argue that the normative foundations for why vaccine-producing companies *should* engage with COVAX seem sound (from a PCSR perspective), empirical knowledge of what companies actually do in such situations is limited. With our analysis, we aim to help close this research gap. We also contribute to knowledge on how the vaccine-producing companies actually behaved during the midst of a global pandemic, thus shedding light on the question of whether these companies—during a global health crisis—lived up to the political responsibility of helping to create equitable access to Covid-19 vaccines. We argue that our investigation of pharma companies’ inclinations to engage with COVAX provides a good opportunity to contribute to the general debate on the motivations of companies to live up to their purported political responsibilities as advanced by PCSR scholars.

## Method

Semi-structured interviews with key stakeholders were combined with archival data sources and a systematic analysis of media reports to provide a comprehensive investigation into the factors influencing pharmaceutical company engagement with COVAX. The data were collected in a “snapshot” period from November 2020 to early May 2021, during the development of the pandemic and evolving company responses.

We began with openly accessible documents (i.e., Gavi board meeting minutes, presentations, and organizational documents) to gain a better understanding of temporal and interactor dynamics in relation to COVAX (Miles et al., 2020). These documents were used to build understanding of the functions of COVAX, the vaccine procurement process, and allocation framework, as well as the timeline.

We then conducted semi-structured interviews online with representatives of COVAX stakeholders (including the Access to Medicine Foundation, Bill and Melinda Gates Foundation, Coalition for Epidemic Preparedness Innovations [CEPI], European Commission, GSK, IFPMA, Johnson & Johnson, Novartis, People’s Vaccine Alliance, Sanofi, Takeda, UNICEF, the United States Agency for International Development [USAID], WHO, and the World Bank). Informants were selected to mirror the diversity of COVAX stakeholder perspectives. In addition, we interviewed two experts who had insight into COVAX through their advisory roles (i.e., as academic experts and consultants). For industry interviews, we targeted companies with vaccine candidates that had received WHO emergency approval before May 2021 (as noted, the WHO approval was essential because only approved vaccines could be purchased and distributed through COVAX).^10^ This was an exceptionally busy time for everybody associated with COVAX. We were fortunate to be able to obtain 21 interviews with participants representing the stakeholder categories shown in Table 1 (including four interviews with three vaccine-producing companies). To ensure anonymity, we assigned codes to interviewees based on their stakeholder group. The research team transcribed a total of 13 hr and 29 min of recorded material, resulting in 193 pages of single-spaced interview transcripts.

**Table 1.** Overview of Interviewees.

Interview	Group	Position	Group description
Bus1	Business	Head of Global Vaccines Program	Companies in the pharmaceutical industry and pharmaceutical industry representation groups.
Bus2	Business	General Director	
Bus3	Business	Public Affairs Head	
Bus4	Business	Head of Global Market Access	
Bus5	Business	Vice President of Global Program	
Bus6	Business	President of Global Vaccines Program	
Bus7	Business	Head of Global Policy Strategy	
Bus8	Business	Head of Global Public Health	
CSO1	Civil society organization	Researcher to the Executive Director	Civil society organizations focusing on access to medicine
CSO2	Civil society organization	Advisor	
E1	Expert	Member of a COVAX Task Force	Experts regarding the planning, establishment, and operations of COVAX.
E2	Expert	CEO and Founder of a consultancy firm in the health care sector	
FM1	Funding member	Senior Health Economist	Organizations that fund COVAX activities and are involved in decision-making
FM2	Funding member	Head of Development and International Cooperation	
FM3	Funding member	Deputy Director, Office of Health	
FM4	Funding member	Director, Health Products, Programs, and Markets	
IN1	Institutional member	Director and Strategic Advisor to the CEO	Organizations that have led the setup of COVAX as well as ensured operational functioning
IN2	Institutional member	Deputy Director Of Operations	
IN3	Institutional member	Director, Vaccines	
IN4	Institutional member	Director of Business Development	
IN5	Institutional member	Covid-19 Vaccine Access Coordinator	

*Note.* COVAX = Covid-19 Vaccines Global Access Facility; CEO = chief executive officer.

Following each interview, emerging themes were discussed and summarized by the research team. Thereafter, each interview was transcribed, and more formal coding procedures were employed (Miles et al., 2020). Interview transcripts were coded by two members of the team, following the template analysis approach (King et al., 2018; Knights & Clarke, 2014). Preliminary coding was carried out for a subset consisting of 10 interviews. The resulting codes were discussed among the two coding researchers to cluster them into overarching themes (King et al., 2018). These themes were derived from the clustered codes across the qualitative data set.

When disagreements on the clustering of codes arose, the researchers discussed the matter until agreement was reached and an initial template could be created. Subsequently, the remaining interviews were coded in an iterative process of moving between our themes and the data, expanding and amending themes and codes when necessary. Following the data collection stage, the final set of identified themes and the relationships between them were discussed to ensure homogeneity of each category and a shared understanding between the researchers.

In parallel, we conducted an exploratory media analysis using the Factiva database to better understand the timing of company engagement with COVAX, and to compare our interview findings with media reports. The terms “ACT-A OR ACT-Accelerator OR CEPI OR COVAX OR Gavi” were used to systematically search for relevant news articles. Following approaches applied in the extant literature (Hoffman & Ocasio, 2001; Kulchina, 2014), the search was restricted to English-language articles published between March 1, 2020, and March 1, 2021, from four leading and high-circulation English-speaking business outlets: *Financial Times, Wall Street Journal, Forbes Magazine*, and *Economic Times of India*. High-circulation business publications act as opinion leaders and thereby influence the media coverage of other outlets, providing a quasi-representative perspective on the wider press discourse (Bednar, 2012). In addition to our Factiva search, two prominent industry publications, *Fierce Pharma* and *Pharma Times*, were also included (using the same search criteria) to obtain more industry-specific data.

Overall, 1,523 articles were downloaded. After removal of duplicates and articles that did not meet our inclusion criteria, 114 articles remained. To be included, articles needed to discuss COVAX in detail, discuss COVAX in relation to pharmaceutical company engagement, or outline facilitating and/or hindering factors for company involvement with COVAX. The media articles were coded openly and summarized based on reoccurring topics. The most common categories were vaccine nationalism (discussed in 48 articles); criticism of high- and middle-income countries (30); geopolitical tensions due to Covid-19 (21); bilateral manufacturing collaboration within the pharmaceutical industry (16); and insufficient funding and slow setup of COVAX (15). As a final step, we combined the identified themes from the interview data with our codes from the media analysis. This was done by clustering the codes from the media analysis and integrating those thematic clusters into our template.

## A Thematic Account of Company Engagement With COVAX

**Figure 1.**
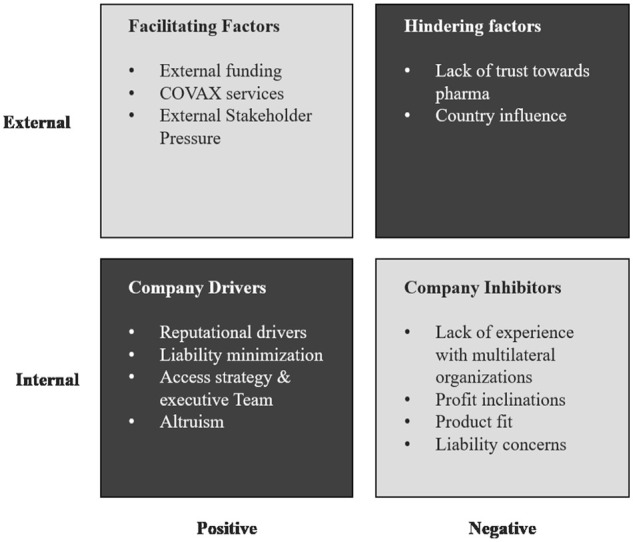
Factors Influencing Pharmaceutical Company Engagement With the Covid-19 Vaccines Global Access Facility (COVAX).

Our interviews and media analysis generated empirical findings on the factors motivating companies to engage with COVAX. We structure these findings according to whether they are *market-based* or *political-institutional* reasons. In doing so, we conceptualize market-based explanations and political-institutional explanations to be two ends of a spectrum along which the individual themes are situated; with some being clearly on one end, while other themes may be situated closer to the center (Bartley, 2007; de Bakker et al., 2019; Pacheco et al., 2010). These factors may either facilitate or impede pharmaceutical company engagement with COVAX and are either internal to the company or external (see Figure 1). Our analysis also identifies *moral considerations* that may help explain the observed differences in degree of company engagement with COVAX. We discuss each in turn and include references in parentheses to the relevant interview and media sources (see Table 1 and appendix).

### Market-Based Reasons

Market-based explanations suggest that companies engage with a multistakeholder partnership (COVAX in this instance) out of enlightened self-interest. This would be to *seize business benefits* (e.g., strengthening their market position, increasing profits, or improving their reputation; Ordonez-Ponce et al., 2021; Tashman et al., 2022); *reduce risks* (e.g., reputational risks, market risks, or legal risks; de Bakker et al., 2019; Fransen & Burgoon, 2014); or *respond to external stakeholder pressures* (e.g., investor pressures, consumer demands; de Bakker et al., 2019; Vurro et al., 2010). Our investigation of company engagement with COVAX lends support to these established explanations, which we now develop and explain in more detail.

#### External Funding

By participating in COVAX, vaccine-producing companies could seize financial benefits by gaining access to external R&D funding through CEPI, as well as “secure[d] financing and streamlined procurement” processes [IN4]. Thus, the financial risks incurred in vaccine development and manufacturing at risk, before a vaccine’s approval by health authorities, could be lowered. As another interviewee confirmed,

having funds enables them to complete the work of actually bringing a product into existence through the regulatory approvals process. It’s pretty significant (. . .) for the smaller companies that don’t have the kind of capital that larger companies do and so that’s a good thing. [IN4]

Funding seemed to be especially important for companies struggling to develop an effective Covid-19 vaccine:

If you look at (. . .) what’s happening in the vaccine market, it’s a land-grab by Pfizer (. . .) So, what is Pfizer’s incentive to help everyone else scale up their production? It’s very low, but if you’re one of the others that’s trying to get up and struggling a bit, then you have a much stronger incentive to collaborate. [E2]

This sentiment was supported by other interviewees as well:

the companies that were supported by CEPI, you know, get somebody to buy their product and get an early commitment to have somebody buy their product when they don’t know if it’s going to work. Yet, the companies that are more established, they figured it’s worth them taking the risk. [IN1]

With an increasing number of iterations and trial rounds, the costs mount and it becomes increasingly difficult for companies to extend R&D phases without external funding. In addition, as our media analysis confirmed, “even profitable big pharma groups have shied away from investing in vaccines for outbreaks without public funding” [FT1].

#### COVAX Services

In addition to funding, COVAX offered several services to participating companies, such as a *streamlined regulatory framework and processes* (which also tie in to opportunity-related company drivers for engagement). This made it possible for companies to access global markets through a single channel, “rather than worrying about regulation in country X and Y and Z and the costs and problems of registration” [CSO2].

Once committed to COVAX, companies could also take advantage of the WHO-established *vaccine allocation scheme*, which meant that companies could delegate morally difficult decisions about who gets the vaccine to an external entity [BUS1, FM3]. As one of our company interviewees explained,

you don’t want to be as a company in the middle of deciding on allocation (. . .) we’re not well positioned to decide which volume should go where or which country needs it most and [COVAX] makes it easier for industry. [BUS1]

COVAX also provided the *logistics and distribution networks* to minimize company risk and optimize operational functionality. Working with UNICEF, through COVAX, companies were able to benefit from its extensive experience in vaccine distribution in developing countries: “the distribution of vaccines is challenging and COVAX has an infrastructure that exists” [FM3]. In relation to this, the media analysis and some interviewees [FM2, BUS1, IN2, FM3, and BUS3] also emphasized the reduced risk associated with COVAX-managed logistics and supply chains. Companies could take advantage of established vaccine distribution platforms and rest assured that “it’s a machinery which works already. You know it has proven to work when it comes to vaccine distribution” [FM2].

In sum, COVAX provided *market access* [CSO1, FM2, BUS1, CSO2, IN1, IN2, IN3, and IN4]. On one hand, COVAX could “ensure that vaccines are accessible to these [low and lower-middle income] countries at affordable prices” [FM2] and companies could “make [their] vaccines available to end this pandemic (. . .) in the global market place” [IN1]. While especially relevant for companies with less vaccine experience, established manufacturers could also benefit from the “opportunity to have access, [the] right, to these markets that otherwise would be very difficult for them” [IN2], as we have explained.

#### Reputational Drivers

Consistent with the literature on marked-based approaches, our interviewees mentioned factors concerning company *reputation* as an essential decision factor. For one, companies strived to *protect their public image* as responsible companies. Although engagement with COVAX did not influence company rankings in the Access to Medicine Index (an independent ranking based on pharmaceutical company efforts to improve access to their medicines in poorer countries), interviewees reported a general industry awareness of the link between increasing access to medicines and company reputation [E1, CSO2, CSO2, IN3, and E2].

Some companies also viewed engagement with COVAX as an *opportunity to strengthen reputation*. However, this opportunity was greater for lesser-known companies or companies not traditionally active in vaccines, whereas established vaccine-producing companies with sufficient financial means were more reluctant to engage because they “do not want strings attached” [IN1]. Some companies tried to leverage their COVAX engagement publicly to improve their reputation [CSO1, CSO2, IN1, BUS1, and BUS2]. Johnson & Johnson and AstraZeneca strongly emphasized their commitments to COVAX in major communications relating to their vaccine candidates [E1 and FM2]. While for “the lesser-known companies and candidates (. . .) it’s a way to sell vaccines and to make vaccines available to end this pandemic as well as to get a lot more brand recognition in the global marketplace” [IN1].

Obtaining access to global markets through a single channel (COVAX), new market entrants were able to capitalize on international demand for limited supplies, while also gaining reputational benefits from helping to provide vaccines equitably for low- and lower-middle-income countries during the crisis. One business interviewee spoke about how company action on access to medicines contributed significantly to a positive reputation with existing and future employees [BUS8]. Moreover, after most high-income countries had secured direct deals with the initially successful vaccine producers (e.g., Pfizer), companies that missed out joined COVAX because “they go towards what’s left, but at the same time they go for what’s good for their reputation. And then they look like the good guy” [CSO1].

#### Liability Minimization

While business opportunities were important, our interviewees suggested that companies placed a greater premium on the opportunity to minimize *legal liabilities* [FM2, IN1, BUS2, IN3, IN4, and FM3]. Several interview partners reported that companies may have engaged with COVAX to *minimize their liability* because COVAX offered both an established indemnification and liability mechanism [IN4] and a “no-fault compensation scheme” [IN3], which was especially relevant for distribution in countries potentially unable to meet the terms of the indemnification [FM2, IN1, BUS2, IN3, IN4, and FM3]. As an institutional member explained, COVAX is

addressing industry’s concerns around liability through indemnification [agreements] and then also this compensation mechanism; [this] is something that is considered by industry to have been well done and effective and therefore a separate inducement for them to participate in supplying their products through COVAX because, you know, they have that degree of comfort in terms of the introduction of vaccines into markets, where there may indeed be adverse events arising. [IN4]

The no-fault compensation fund for the GAVI-92 countries (the 92 low- and middle-income countries [LMIC] covered by the Advanced Market Commitment in COVAX) was created because some countries might not be able to live up to the terms of the indemnification: “It really sort of de-risks for industry, a lot of the legal liability, and obviously makes it much fairer to compensate people in these countries in the event of harm from the vaccine” [IN1].COVAX also ensured that the no-fault compensation scheme was fully funded, providing financial security to LMICs potentially unable to pay out liability claims [BUS3]. In addition to our interviewees, the media analysis also suggested that liability issues were a major concern for companies, exacerbated by the fact that standard liability insurance was not available in the pandemic context.

#### External Stakeholder Pressure

When it comes to the “stakeholder pressure” factors identified in the literature as part of market-based reasons, multiple different mechanisms seem to be at play with COVAX. On one hand, investors, the media, state actors, as well as civil society representatives and international organizations were exerting pressure on vaccine manufacturers. On the other hand, media reporting (and criticism) focused more strongly on the role and responsibility of high-income countries in striking direct deals with vaccine manufacturers and much less on their counterparts, the pharmaceutical companies.

Moreover, even though investors had increased their attention to the access-to-medicine issue recently [CSO1], this did not appear to directly inform company Covid-19 vaccine access strategies. Nonetheless, investors, civil society representatives, and the media exhibited support for COVAX and exerted pressure on vaccine manufacturers to participate: “there was a hell of a lot of pressure from civil society, the public, the media” [CSO2]. Another interviewee commented that there was action “only really when we were ratcheting up the pressure and [. . .] saying, ‘we’re going to have to start saying something about why there’s no deal’” [IN2].

The stronger the external stakeholder pressure on companies, the more difficult it became for them not to participate in COVAX to avoid “negative press” [E2]. As one interviewee described it,

Pfizer committed 40 million to COVAX, so then again never too late (. . .) but then again, they already had their fair share of high-income countries and money there, right? (. . .) it’s all a strategy game for them, it’s how they can be seen in the eyes of the public, how they can be seen in the eyes of the governments, and how much money they can make. [CSO1]

Moreover, external pressure was not one way: “those investors will want to see a return” [E2]. However, “Covid (. . .) put access to medicines (. . .) as a material issue for investors on the map” [CSO1].

Interviewees also reported that vaccine manufacturers were exposed to various *political pressures* [CSO2, IN2, and IN5]. Some spoke of the perennial threat from intellectual property waivers (Vachani & Smith, 2004), if companies did not engage with COVAX. Meanwhile, vaccine-producing countries such as India, Russia, and China (where governments were in control of national vaccine manufacturing) appeared to follow geopolitical motivations in their Covid-19 vaccine distribution strategies, engaging in a form of “vaccine diplomacy” [ET22]. These governments pressured manufacturers in their countries not to engage with COVAX, so that vaccine allocation could be used instrumentally to “bolster (. . .) international image by increasing support for global health initiatives” [FT17].

#### Lack of Previous Experience With Multilateral Organizations

In addition to these market-based drivers, we found a range of market-based inhibitors to company engagement with COVAX. Even if companies showed strong interest in engaging with COVAX, some experienced difficulties due to their *lack of experience with multilateral organizations* in collaborating with global organizations such as Gavi and UNICEF [IN3]. As one interviewee explained,

We started and then we just weren’t able to come to a final larger agreement and it wasn’t for lack of trying, and I don’t think it was for lack of any good faith on Moderna’s part (. . .) it was really early days. [. . .] (. . .) I know individual people in the company that had come from other companies that had engaged in this, but as a company collectively, they hadn’t pulled that kind of stuff together. [IN4]

Moreover, collective action is not common in pharma. As one interviewee puts it, “sometimes they do collaborate on certain fronts. But most of the time I want to say this is not current (. . .) practice in the industry” [CSO1]. Companies generally engage in “bilateral manufacturing arrangements” [BUS4] and technology transfers [CSO1]. The media analysis also indicated that if pharmaceutical companies did collaborate, it most commonly took place bilaterally; for example, media articles elaborated extensively on the number of manufacturing agreements struck by AstraZeneca to increase global vaccine production capacity and supply.

Lack of experience in vaccines also potentially led to an “underestimation of complexity” [BUS1], as well as an overcommitment by some pharmaceutical companies [E2]. It also created communication hiccups for some companies [BUS1 and E2]. As one interviewee (from a different pharmaceutical company) puts it (also see related media coverage [FP10]),

If we look at AstraZeneca as an example, who is also not an experienced vaccine company, I think. They probably, well definitely, stepped into this with all the big, I would say, goodwill. And then I think, because of lacking the experience, I think probably did not realize the difficulty in which it could end up in all of these supply discussions, because, of course, with experience, you know that you will have lots of manufacturing issues and that you know scaling up is a challenge and that you need to be careful on what you promise because difficulties will arise. [BUS1]

#### Product Fit

Interviews and media analysis indicated some hesitation by COVAX toward certain vaccine candidates, reflecting a lack of product-market fit [E1, FM2, IN2, E2, and BUS8]. Building on the general viability question of mRNA vaccines (as further discussed in the opportunities and company strategy section), experts were additionally skeptical toward the appropriateness of mRNA vaccines in developing countries. These concerns mainly stemmed from the *ultra-cold chain logistical setup* and the increased supply chain difficulties:

So, this explains why (. . .) [Gavi] didn’t conclude deals for that huge number of doses, because of the ultra-cold chain requirements which are difficult to handle in some of the 92 countries (. . .) the infrastructure is not there. [FM2]

High-priced vaccines were also deemed less appropriate for low- and lower-middle-income countries [FM2, IN2, IN4, and BUS8]. And while COVAX had created a no-fault compensation scheme (discussed earlier), some companies still had liability concerns and required additional indemnification and liability agreements as well as country-specific checks before delivery [FM1]. This was particularly a concern for those manufacturers with low product-market fit for their vaccines in developing countries. Media articles as well as interviewees elaborated on the heightened risk stemming from the distribution of vaccines requiring the ultra-cold chain distribution [E1, FM2, IN2, and E2], aside from the broader concerns of administering vaccines that only have emergency use authorization [IN5] and in a pandemic of unprecedented scale with many unknowns.

#### Profit Inclinations of Companies

Ultimately, company engagement with COVAX was still inhibited by the common denominator of profit, the exigencies of a pandemic notwithstanding. Some vaccine-producing companies did not engage or engaged only to a modest degree because of their profit inclinations [CSO1, FM1, FM2, BUS1, CSO2, IN1, BUS2, IN2, IN3, IN4, E2, FM3, and BUS8]. From an opportunity cost perspective, especially in the early phases of vaccine production and distribution when global supply of vaccines was limited, it was significantly more profitable for companies to cut direct deals with richer nation-states or the European Union (EU) than to engage with COVAX. Vaccine manufacturers were dealing with a trade-off between “commercial opportunity vs. contribution to public health” [BUS1]. It was acknowledged that companies took operational and financial risks when creating vaccines, but did that justify “pay[ing] most attention to the demands of those with [the] deepest purses? (. . .) Such realities cannot be ignored. But can they be transcended?” [FT51].

### Political-Institutional Reasons

While our results demonstrate the importance of market-based drivers and disincentives, they also indicate that company engagement with COVAX can be explained in part by reasons that are more *political-institutional*. While political-institutional approaches acknowledge that companies mostly seek to advance their self-interest, they also conceive of companies as embedded in their social and institutional context—including societal expectations of responsible business conduct—to which they need to adapt to maintain legitimacy (Bartley, 2007; Dashwood, 2014; de Bakker et al., 2019; Reinecke & Ansari, 2016).

#### Lack of Trust Toward Pharma

While COVAX created incentives for vaccine manufacturers to join, pharma was still mistrusted within some COVAX member organizations and stakeholders. The industry had improved its image over the past decade (Edelman, 2020), but it started from a low base and doubts were expressed about the motives of pharmaceutical companies, leading to factors disincentivizing engagement with COVAX [FM1, BUS1, IN1, IN2, and BUS4]. One company representative commented the following: “we are not really seen as a 100% trusted partner (. . .) there is always this (. . .) assumption that industry will go for profits before going for (. . .) the right solution or the best solution” [BUS1]. Some actors within COVAX were opposed to including industry representatives on committees, which made collaboration with the industry more difficult:

They didn’t want a current industry person and they didn’t want anybody representing one of the industry organizations and it was hard to settle on and identify a former industry person who could speak from the industry perspective (. . .) that’s clearly something that desperately needs to be fixed going forward. [IN1]

#### Country Influence

The media analysis as well as our interviewees highlighted how governments impeded the initiative in multiple ways. They included insufficiently timely monetary commitments to COVAX from high-income countries and, critically, the countries’ bilateral, or in the case of the EU, supranational deals with vaccine-producing companies as an alternative to using COVAX. This emerged as the major obstruction to company engagement with COVAX, identified by all interviewees as well as throughout the media. The bilateral national- and EU-level deals led to a situation where vaccines were no longer available in sufficient quantities when COVAX was eventually operational, equipped with political legitimacy, and (at least) initial financial resources with which to buy vaccines. This problem was also referred to as “vaccine nationalism” [BUS3, IN3, and IN4]. As one interviewee explained,

there are certain countries that are very, very, very advanced on their vaccination program. What happened was that they bought the vaccines in advance of everybody else (. . .) so they stockpiled, and they ensured that their markets were (. . .) not only fully served, but in certain cases, they secured vaccines to vaccinate their populations three times over. [IN2]

Vaccine nationalism was also by far the most identified theme in the media analysis, not only how it created unnecessarily heightened levels of competition and supply shortages [e.g., FT6, FT57, F8] but also how it limited the ability to quickly scale the production of the best vaccine candidate [e.g., F7, WSJ11].

Vaccine nationalism was especially perilous for global health when countries (e.g., India) imposed export bans on vaccines (and their ancillaries) so as to direct production to serve their domestic populations [FM1, IN3, E2, BUS3, CSO2, and BUS2]. Media articles drew parallels with government behaviors and business practices exhibited during the H1N1 outbreak in 2009 and urged that the “response to the pandemic does not have to copy the failures of swine flu” [FT11]. Similarly, interviewees emphasized that Covid-19 vaccination was a global issue and that “no one is safe until everyone is safe” [BUS2], a point also widely reflected in media articles: “Businesses and governments must understand that the future is not a zero-sum contest in which winners win only when someone else loses. It is a co-operative endeavour in which we all make progress together” [FT13]. Nonetheless, the “highest-bidding” [CSO1] industrialized countries that secured a quick supply of vaccine doses for their own populations did promise to donate excess doses to poorer countries, with excess supplies to be potentially traded on a COVAX exchange platform [FM3 and IN2].

Also weakening COVAX was some countries not wanting the AstraZeneca vaccine because it was considered less effective against the SARS-CoV-2 variants dominant in their region [E2 and WSJ10].^11^ Some countries were hesitant about joining COVAX for geopolitical reasons and preferred to purchase vaccines outside of COVAX [IN2]. Equally, some countries were skeptical of COVAX’s effectiveness and sought alternative arrangements, such as the African Union’s vaccine pool [FM1 and BUS2]. Accordingly, the media analysis highlighted articles that argued for procurement alternatives to COVAX, aiming to ensure vaccine supply in LMICs.

Regulatory threats (such as debates on IP waivers) seem to have played only a minor role in company engagement with COVAX as of mid-2021, perhaps because no concrete regulatory threats had become manifest during the first year of the pandemic. This issue became more prominent in the second half of 2021.

### Moral Considerations

Given that most of the market-based and political-institutional factors apply to all pharmaceutical companies to a certain extent, these factors cannot fully explain the differential engagement with COVAX. *Micro-level moral factors* at the executive and company level also appear to have played a role.

#### Access Strategy and the Executive Team

We found that company engagement with COVAX was also driven by the inclinations of the executive team and the associated commitment to generating access in their corporate strategy. One interviewee described companies as “hav[ing] personalities” [IN3] reflected in corporate strategies and values, and multiple interviewees referred to corporate culture and strategy as a relevant factor in company decisions to engage with COVAX early on [BUS1, CSO1, E2, and FM3]. Some companies had made increasing access to medicine an important part of their strategy in recent years [BUS1 and BUS8] and one interviewee commented the following: “we see the pharmaceutical industry move in the right direction on access when there is a clear prioritization of the needs” [CSO1]. Nonetheless, performance in the Access to Medicine Index does not appear to predict the company level of engagement with COVAX (see Online Appendices for further information).

Commitment to global equitable access to medicines and engagement in COVAX was reportedly facilitated by the inclinations of individual executive team members [E1, CSO1, IN1, IN2, IN3, and E2]. “There are a bunch of candidates that were not [financially] supported by CEPI, where (. . .) the CEO feels strongly, a social and global obligation, like J&J” [IN1]. However, the strategy of some companies with regard to COVAX was said to be detrimental to some company decisions to engage with the initiative [E1, CSO1, and IN1]. The difficulty in dealing with companies that were not willing to substantially engage with COVAX is described in detail by one interviewee representing an institutional member. Noting that “there have definitely been manufacturers who are very clear from their corporate perspective that they are in this for an understanding of their contribution to ending a pandemic,” this person continued:

There are other manufacturers who are very clear that they are in this from a business perspective, and so have been unwilling to actually have any serious conversation about tiered pricing (. . .). Companies who have (. . .) put one barrier after the next in front of actually deploying vaccines (. . .). Like, there are definitely companies where nothing was good enough and wanted to drill down on absolutely every single thing, while simultaneously there were companies that were like, “yeah, this is fine, let’s get going.” (. . .) Some manufacturers (. . .) have been really, really hard to work with. [IN3]

While unwilling to state it directly, this interviewee essentially suggests that some companies hampered negotiations with COVAX to delay supplying vaccines that could be more profitably sold elsewhere (see section “Understanding the Differential Engagement of Companies With COVAX”).

#### Altruism

Inclinations of the executive team seemed to be closely associated with the individual altruistic motives and internal moral drivers for COVAX engagement, as several interviewees reported. Underlying reasons to engage with COVAX were described by company representatives as well as external stakeholders as wanting to “do the right thing” [E2] and to ensure a need-based allocation to “contribute to global good” [FM3]. It was also portrayed as being about having a corporate strategy that does not put profits first [CSO1, FM2, and BUS1].

As one of the main roles of COVAX was to deal with allocation issues, lifting the burden from companies of making moral decisions on the prioritization of vaccine access was likely another reason behind company engagement. While still cutting deals with individual countries, “firms do not want or cannot decide on the global allocation processes” [BUS1]. Meanwhile, the WHO’s Dr Tedros charged that, “It’s not right that younger, healthier adults in rich countries are vaccinated before health workers and older people in poorer countries” and he called on pharmaceutical companies to actively participate in equitable global access [FT37].

## Understanding the Differential Engagement of Companies With COVAX

Companies differed markedly in their degree of engagement with COVAX. To understand why, we take three of the leading vaccine-producing companies as exemplary cases (AstraZeneca, Pfizer/BioNTech, and Moderna). While our research certainly indicates a role for *moral considerations*, companies primarily engaged with COVAX because of *market-based* and *political-institutional* factors. Applying our prior analysis, we argue that differential *contractual obligations, opportunities and company strategy*, and *reputational pressures* are the factors that best explain why these firms engaged with COVAX to a varying degree.

### Funding and Contractual Obligations

The individual engagement of vaccine producers has to be understood in light of their funding sources and contractual obligations. There were substantial differences in the extent to which the three companies accepted public funding for vaccine development. Contractual obligations tied to funding by countries significantly influenced companies’ vaccine access strategies and their ability to engage with COVAX.

While CEPI funded various vaccine candidates, including those of Oxford University and Moderna (CEPI, 2021), the U.K. government provided substantial funding for AstraZeneca (Safi, 2021) and the U.S. government-funded Biomedical Advanced Research and Development Authority (BARDA) also provided significant financial contributions to help fund the research of Moderna and AstraZeneca (BARDA, 2021). National financial contributions were commonly tied to first-access contractual clauses, limiting the scope for firms to engage with COVAX from the outset (for Moderna, see Moderna, 2020; for AstraZeneca, see Isaac & Deutsch, 2021). Pfizer received public funding indirectly because its partner BioNTech was heavily subsidized by the German government for many years before Covid-19 (Griffin & Armstrong, 2020). However, Pfizer rejected direct funding from BARDA. Thus, it was not bound by contractual obligations and was able to maintain full control of its vaccine distribution strategy (Czachor, 2020). By contrast, AstraZeneca faced the strongest pressure to engage with COVAX, due to the funding it had received from CEPI, as well as the contractual commitment made to Oxford University to provide equitable global access. However, due to the bilateral agreements it had struck with the U.K. government, AstraZeneca prioritized supplying the United Kingdom during the initial phases of vaccine distribution (Isaac & Deutsch, 2021). While Moderna had received initial R&D funding through CEPI, requiring formal dose commitments to COVAX, the company did not commit to COVAX until 5 months after its initial Food and Drug Administration (FDA) authorization. This was essentially due to the heavy funding Moderna had also received from BARDA, putting it under pressure to serve the U.S. market once its vaccine was authorized (Sagonowsky, 2020).

### Opportunities and Company Strategy

As our interviews and media analysis suggest, COVAX’s institutional members, in line with the predictions made by global experts (Hopkins et al., 2021), severely underestimated the viability and/or scaling capacity of mRNA vaccine programs (e.g., CureVac, Moderna, Pfizer/BioNTech). Against all expert predictions, not only mRNA technology vaccines were authorized for the first time in history in 2020 but also Pfizer’s Covid-19 vaccine was the first vaccine candidate to become available. The unanticipated quick development and the timing of authorizations by multiple national health authorities gave Pfizer (in cooperation with BioNTech) a significant competitive (first mover) advantage in satisfying the immense global demand (Lieberman & Montgomery, 1988). We suggest that Pfizer’s behavior relative to COVAX can best be explained by a market-based rationale (i.e., its actions were principally motivated by instrumental reasons). Pfizer maximized its profits by supplying vaccines to the highest bidders through direct deals (Robbins & Goodman, 2021; UNICEF, 2021). Adding to this, Pfizer arguably had much less to gain from engaging with COVAX as an established player in the pharmaceutical industry with extensive expertise in vaccine manufacturing and global distribution. Pfizer could better ensure vaccine quality and limit the associated liability concerns by remaining in control of vaccine distribution and forgoing (substantial) COVAX participation.

AstraZeneca’s approach to vaccine distribution must be understood quite differently, but still as at least partially instrumental. Given its pledge to a not-for-profit strategy, the company initiated discussions with COVAX in mid-2020 and came to terms on dose commitments as early as June 2020. Considering that AstraZeneca’s vaccine candidate was provided by Oxford University under a contractual commitment to nonprofit pricing and global access, AstraZeneca was strongly incentivized to engage with COVAX. However, dealing with COVAX was also a volume-maximizing strategy for the company that secured substantial revenues and the prospect of future profits with the end of the pandemic (when it could drop the not-for-profit provision in many markets). This strategy is also evident in the large number of bilateral manufacturing and technology transfer agreements that AstraZeneca struck globally to increase production capacity (Reuters, 2021).

By mid-2021, Covid-19 vaccine demand in high-income markets had largely been met through direct deals. This encouraged Moderna to consider alternative channels of distribution. According to some of our interviewees, this was a further explanation for why Moderna committed to COVAX relatively late—to gain access to global markets [E2]. Due to the company’s limited experience in vaccines, Moderna could also realize significant benefits from COVAX services, as previously described.

### Reputation

Our interviewees highlighted that the vaccine-producing companies were exposed to varying reputational pressures from the media, governments, civil society, as well as industry representatives, which substantially influenced their Covid-19 pandemic response.

These reputational pressures can also help to explain Pfizer’s engagement in COVAX. As an established player in the global vaccine business, which had publicly advocated for equitable global access and had been ranked highly in the Access to Medicines Index over the preceding years (Access to Medicine Foundation, 2021), not engaging with COVAX could have been perceived as hypocritical. As one of our interviewees indicated, by committing a small number of vaccines to COVAX, Pfizer largely avoided negative press (as of mid-2021) [E2].

AstraZeneca turned its contractual obligations to Oxford University—alongside its difficulties in securing FDA approval and U.S. market entry—into an opportunity to reap reputational benefits from an access-focused vaccine distribution strategy. Its extraordinary commitment to global access received significant praise, with some going as far as proclaiming it was the “white knight” in vaccines [BUS7]. However, AstraZeneca also faced significant public relation challenges. It stumbled in its communications, facing criticism over R&D reporting of vaccine efficacy data, dose supply, and potential side effects—problems attributed to the company’s lack of experience in vaccines (Boseley, 2021). Delivery delays, difficulties in meeting vaccine supply commitments, and the associated communication missteps escalated into legal action being taken by the EU against the firm (Guarascio & Vagnoni, 2021).

Moderna is somewhat of an outlier because it had not launched a commercial product before its success with the Covid-19 vaccine. It therefore did not have an established reputation in the industry. Interviewees suggested that Moderna was exposed to higher levels of investor pressure to maximize revenue than its counterparts with successful vaccine programs [E2 and BUS8]. They believed that this had reduced the incentive for Moderna to engage with COVAX. However, after the demand in high-income markets had been saturated, our interpretation is that the company could then approach the distribution of its vaccine more strategically, to build a positive brand image by supplying COVAX.^12^

## Discussion and Conclusion

Our investigation of corporate practices during a global health crisis explores a profoundly important business and society relationship. We examine how companies engaged with COVAX, a vital global institution in the context of the Covid-19 pandemic. At a general level, our research contributes to a better understanding of the crisis responses of business within the broader societal context (see Bapuji et al., 2020), by identifying key hindering and facilitating factors for company engagement with COVAX (see Figure 1). More specifically, our investigation contributes to the debate on PCSR, adding some nuance to the question of whether MNCs would live up to their responsibilities in the sense of providing help in addressing global issues that nation-states seemed unable to adequately address. In so doing, it also speaks to multistakeholder partnerships and the issue of access to medicines.

### Contributions to Theory

The results of our investigation overall suggest that the engagement of vaccine-producing companies with COVAX was indeed driven *primarily* by instrumental factors. These results lend prima facie support to the position of the PCSR skeptics (Rhodes & Fleming, 2020; Whelan, 2012). However, the situation is more nuanced than it might appear. Market-based approaches alone are insufficient to explain pharma engagement with COVAX. This conclusion is consistent with de Bakker and colleagues (2019), who argue that an exclusive focus on an instrumental business case logic is insufficient in explaining company adoption of Metabolomics Standards Initiative (MSI) standards.

The *political-institutional perspective* helps us understand why *no* company with a viable Covid-19 vaccine could afford to ignore COVAX entirely. Acting contrary to societal expectations would potentially jeopardize their social license to operate (Access to Medicine Foundation, 2021; Demuijnck & Fasterling, 2016; Leisinger, 2009). Access to medicine has become a prominent issue (Leisinger, 2009) and stakeholders including investors now pay close attention to performance on this dimension. Moreover, as the number of companies engaging with COVAX increased, isomorphic pressures on the remaining companies arguably also increased, to which they responded by ultimately engaging as well. After several companies had pledged millions of vaccine doses, engaging with COVAX had become the norm.^13^

We also suggest that *moral considerations* are relevant to explaining company behavior. Our interviewees certainly highlighted moral considerations as a factor in AstraZeneca’s response to COVAX [e.g., IN3]. More in this regard has emerged about the Oxford University and AstraZeneca collaboration subsequent to our empirical research (The Economist, 2021c; Walsh, 2022). The Economist (2021c) reported that “Pascal Soriot, AstraZeneca’s boss, has always insisted the decision to make the vaccine was fundamentally altruistic rather than commercial (. . .).” While this narrative has rhetorical appeal, our analysis suggests that moral considerations are only part of the story. In our investigation, we identified moral factors at the meso- and micro-level that influenced company engagement decisions. Some pharmaceutical companies have made it a central part of their strategy and culture (meso-level) to contribute to fair global access to medicines (e.g., Novartis, Johnson & Johnson). Micro-level CSR approaches—in addition to instrumental reasons—are also relevant to understanding the differential engagement of companies with COVAX (Acosta et al., 2021; Maak et al., 2016).

Various interviewees reported that the inclinations of chief executive officers (CEOs) and executive teams toward the issue of global equitable access and corporate responsibility made a significant difference. As one interviewee stated, “I think there are examples where it took CEOs of manufacturers getting involved with their own team to say, ‘make this damn thing happen’” [IN2]. A business representative highlighted that the “commitment to global health” by some pharmaceutical industry executives “is incredible,” ultimately driving their company’s commitment to equitable vaccine access [BUS6]. We conclude that the moral convictions of individual managers toward global health have been an important factor in the engagement of some companies with COVAX. These findings also contribute to explanations of why companies engage in PCSR more generally, supporting existing theory in suggesting that the moral inclinations of business leaders play an important role in whether and how companies engage with PCSR (Acosta et al., 2021; Maak et al., 2016).

Our research also highlights an important nuance in understanding the role of government in relation to PCSR. Unlike many of the scenarios of weak governments painted by PCSR proponents, governments were very much active and strongly engaged throughout the pandemic, but there was a governance gap at the global level. The failure to provide more equitable access to vaccines via COVAX, the vehicle expressly created to serve this need, reflects an absence of willingness by governments to look beyond their immediate national self-interest. In some ways, vaccine-producing companies might be said to have been responsible at a country level—meeting their responsibilities in responding to demands for vaccines from country governments (especially their own)—but not responsible at the global level. This points to the importance of PCSR theorizing around when companies might be expected to act on grand challenges (such as a pandemic), relative to the part played by governments, who might be able but unwilling to act.

### Normative Implications: Can Society Rely on Companies When They Are Needed?

Our analysis also speaks to normative implications of PCSR. Inequitable access to lifesaving vaccines is an issue of global fairness with human rights implications (Bachelet, 2021). COVAX was created to alleviate this unfairness by providing vaccines to countries with insufficient financial means. Thus, we now return more directly to our core question: *Have pharmaceutical companies (as political actors) lived up to their responsibilities as corporate citizens and fulfilled the implied responsibility of fighting inequitable vaccine distribution?*

Individual companies differed markedly in their response. From a PCSR perspective, it is disconcerting to note how little engagement there was by some companies. Equally, we heard relatively few statements from interviewees suggesting companies had an ethical strategy toward global health, though some wanted to do “the right thing” [E2] and “contribute to global good” [FM3], or had a corporate strategy that did not put profits first [CSO1, FM2, and BUS1].

To be sure, our interviewees repeatedly attested that COVAX (during the time of our investigation) was by no means a perfect institution. Nonetheless, our findings demonstrate that most company engagement with COVAX was *not* consistent with an understanding of a company role as a corporate citizen with a political responsibility to help address grand challenges in the absence of sufficient capacity to act on the part of the traditional political actors, nation-states, and global political institutions. It is not that companies failed to engage with COVAX, it is that they could have done so more strongly. A self-understanding of pharma companies as political actors with responsibilities toward global health was not prevalent.

Early in the pandemic, scholars and practitioners called on pharmaceutical companies to address the challenge of fair global access to vaccines and to publicly voice their concern (Scholz & Smith, 2020; Yamey et al., 2020). They could have encouraged state actors to give greater support to COVAX, including the sourcing of vaccines through COVAX instead of bilateral deals. After our data collection, it emerged that the vaccine-producing companies had considerable power relative to country governments—as one extreme example, it is reported that Israeli Prime Minister Benjamin Netanyahu called Pfizer CEO Albert Bourla 30 times seeking to secure additional vaccines (Kuchler et al., 2021). Companies could have used their power to push back against the vaccine nationalism that militated against supply to COVAX. In Pfizer’s case, with sales of its vaccine at US$36 billion in 2021 (44% of its total sales) and profits doubling to US$22 billion on the back of the vaccine (Pfizer, 2022), many might argue that there was scope for greater sacrifice of profit to support more equitable vaccine distribution by supplying more vaccines to COVAX.

Our results thus spur a general criticism of PCSR and its normative background theory, the Habermasian version of deliberative democracy. While neoliberal models of democracy emphasize nation-states as the dominant political actors, deliberative democracy points to a discursive involvement of stakeholders, including corporate actors as corporate citizens in decision-making processes (cf. Risse, 2004; Scherer & Palazzo, 2007, 2011). In this model of democracy, stakeholder participation is essential. The challenges of corporate engagement in discursive processes are beyond the scope of this article, but our research suggests a more basic problem. If companies are unwilling to participate in this form of democracy—because they lack a self-understanding as corporate citizens with political responsibilities or because their CSR is dominated by an instrumental rationale—it is doubtful from the outset that deliberative democracy can serve as an alternative form of governance to address grand challenges.

The failure of companies to engage strongly with COVAX is unlikely to surprise critics of PCSR. As described earlier, PCSR’s critics reject the notion of moralized corporations, “driven by a concern for the public good that goes beyond the selfish calculations of economic actors” (Scherer et al., 2016, p. 273). While we are sympathetic to the normative claim that companies, especially in a transnational setting and a global emergency, should assume the role of political actors with corresponding responsibilities, our findings are discouraging. Even in the biggest global health and social crisis in decades, the most relevant companies did not strongly engage with the global political issue of equitable access to vaccines or demonstrate much of an understanding of being corporate citizens. For the most part, company engagement with COVAX, when not strongly driven by legal obligations, remained largely peripheral and motivated by instrumental reasons, not a sense of moral and political responsibility.

### Implications for Civil Society and Policymakers

Global inequitable distribution of Covid-19 vaccines as of mid-2021 posed a classic collective action problem, in that the self-interested behavior of pharmaceutical companies combined with the self-interested behavior of individual (richer) countries did not result in an efficient and fair vaccine allocation. Policymakers and companies could have learnt from previous displays of vaccine nationalism, such as during the H1N1 pandemic (Milne & Crow, 2020).

Nonetheless, the profit maximization strategies of individual companies in a global emergency have the capacity to jeopardize the reputation of the entire industry (Scholz & Smith, 2020) and could lead to regulatory interventions (The Economist, 2021b), a bad scenario for all pharma companies. For example, unfair access to vaccines further spurred debate on IP waivers, a major threat to the industry (Gurgula, 2021).

A new globally applicable regulatory framework (e.g., sales and purchase regulations for Covid-19 vaccines) could compensate for the lack of market coordination and ensure that groups are vaccinated worldwide according to their priority ranking. However, given the global governance gap—the lack of an institution that could create and ideally enforce hard laws (Eberlein, 2019)—this regulatory framework seems unlikely to emerge.

One solution could be *private governance* by creating standards for business conduct. If the relevant actors collectively agree on effective rules of the game to address the underlying problem, and if the implementation of these rules is sufficiently monitored and sanctioned, they would establish a new level playing field in which the companies could create a positive societal impact without any individual company losing competitiveness (Kobrin, 2009; Lyon et al., 2018). In the context of access to Covid-19 vaccines, as well as future global emergencies, vaccine manufacturers (e.g., via IFPMA) could create a soft law regime by defining industry rules that provide for global fair access to vaccines (e.g., constraints on sales by bilateral deals).

### Managerial Implications for Improving COVAX

Drawing on our research, we make four suggestions for improving COVAX as a primary point of contact for companies: First, initial funding should be secured in advance of potential future pandemics to make COVAX functional immediately following a new outbreak. Second, COVAX should become a “one-stop-shop” for companies to facilitate company engagement with COVAX. Eliminating the need for three individual contracts for R&D funding, vaccine procurement, and dose allocation with separate organizations, these complex structures could be simplified by requiring only a single agreement. Third, processes should be streamlined to reduce the level of bureaucracy, particularly for companies, but also for other actors involved. While our data suggest that companies appreciated COVAX for the various services it provided, they also indicate that companies perceived the institution as too bureaucratic. Fourth, the stakeholder inclusion criteria should be reconsidered—closer contact with representatives of pharmaceutical companies and civil society organizations could help COVAX make more informed decisions about partnerships and agreements with vaccine manufacturers.

### Limitations and Suggestions for Future Research

A strength of our research is that it was conducted in real time as events were unfolding. It is thus less subject to faulty recollections of events or efforts to rewrite history. Nonetheless, this entails inherent limitations. Some companies’ engagement with COVAX shifted during data collection. Also, because it coincided with critical phases of the pandemic, some key stakeholders were unavailable for interview. Even though we worked hard to reach a wide range of relevant stakeholders (contacting 74 stakeholder representatives between February and mid-May 2021), we were (only) able to secure interviews with 21 internal and external COVAX stakeholders.

Nevertheless, building on multiple data sources, we are confident we reached theoretical saturation during data analysis. We believe further research could address some of these methodological limitations by employing alternative data collection processes and analytic approaches. While it was not the aim of our exploratory qualitative investigation to clearly identify causal mechanisms, we hope that our research enables further research to do so. Further research could also explore the triangular business–government–society dynamic in COVAX, which could enrich understanding of the business and society relationship in COVAX engagement and as it informs PCSR theorizing. Given the development of Covid-19 vaccines by a more diverse set of manufacturers subsequent to our data collection timeframe, future research may be able to provide additional insights into differential engagement in COVAX and account for the influence of different governmental regimes.

## Supplemental Material

sj-docx-1-bas-10.1177_00076503231158600 – Supplemental material for Public Health and Political Corporate Social Responsibility: Pharmaceutical Company Engagement in COVAX Click here for additional data file. Supplemental material, sj-docx-1-bas-10.1177_00076503231158600 for Public Health and Political Corporate Social Responsibility: Pharmaceutical Company Engagement in COVAX by Markus Scholz, N. Craig Smith, Maria Riegler and Anna Burton in Business & Society

## Appendix

**Appendix:** Analyzed Media Articles Cited in This Article.^a^

Code	Publisher	Date	Title
FP10	*Fierce Pharma*	February 22, 2021	AstraZeneca’s Indian COVID-19 vaccine partner told to prioritize local supplies: CEO
FT1	*Financial Times*	March 6, 2020	The $2bn race to find a vaccine
FT6	*Financial Times*	May 14, 2020	Why vaccine ‘nationalism’ could slow coronavirus fight
FT11	*Financial Times*	August 25, 2020	Covid vaccine strategy must consider need as well as wealth
FT13	*Financial Times*	September 16, 2020	Vaccine fairness will make us all safer
FT17	*Financial Times*	October 10, 2020	China joins WHO initiative to offer 2bn inoculations
FT37	*Financial Times*	January 21, 2021	Low-income countries trail in global vaccines race
FT51	*Financial Times*	February 17, 2021	The world’s biggest test of co-operation
FT57	*Financial Times*	February 26, 2021	African price for Russia vaccine blunts attacks on ‘unethical’ west
F7	*Forbes*	February 24, 2021	Ghana Becomes First Nation To Receive Vaccines From WHO-Backed Covax Initiative
F8	*Forbes*	February 25, 2021	Yellen Urges World’s Largest Economies To ‘Go Big’ On Stimulus
ET22	*Economic Times*	February 2, 2021	India-made Covishield part of Pakistan jab drive under vaccine alliance
WSJ10	*Wall Street Journal*	February 18, 2021	World News: Delayed Developing-Nation Shots Risk Extending Crisis
WSJ11	*Wall Street Journal*	February 25, 2021	World News: Ghana First to Get Doses Under WHO Plan

*Note*. CEO = chief executive officer; WHO = World Health Organization.

a For full list of analyzed media, see Online Appendix C.

## Notes

1. Tedros was writing at a time when LMICs were predicted to follow a similar trajectory (in terms of Covid-19 infections, hospitalizations, and deaths) to that of the more developed countries whose populations had been exposed earlier to the pandemic. We know now that the pandemic did not follow the expected trajectory in LMICs. Nonetheless, this does not undermine the case, certainly as of then, for equitable global distribution of vaccines.

2. Doh (2015) makes a strong case for the importance of phenomenon-based research in the international business and business and society fields. Similarly, Ployhart and Bartunek (2019) call for researchers to embrace contemporary phenomena (e.g., see Judge et al., 2011; Muller & Kräussl, 2011).

3. The Bill and Melinda Gates Foundation, CEPI, Foundation for Innovative New Diagnostics (FIND), Gavi, The Global Fund, Unitaid, Wellcome, the World Health Organization (WHO), and the World Bank.

4. For a more detailed account of the history of the Covid-19 Vaccines Global Access Facility (COVAX), see Scholz and colleagues (2022).

5. See Online Appendix A in this article’s Online Supplemental Material section for a list of the major producers of vaccines that had received WHO approval as of mid-May 2021.

6. Online Appendix B summarizes the timeline of company engagement with COVAX.

7. Political corporate social responsibility (PCSR) is not an undisputed concept. See the review by Frynas and Stephens (2015) and the critical response to this review by Scherer (2018).

8. For a toolbox to analyze the differential responsibilities of companies to engage with human rights issues, see Smith et al. (2021).

9. See the UN Declaration of Human Rights, article 25 (1948); World Health Organization Constitution, preamble (1946); International Covenant on Economic, Social and Cultural Rights (ICESCR), article 12 (1966).

10. The Sinopharm vaccine received the WHO emergency approval on May 7, 2021.

11. With Brexit as the backdrop, some European politicians also spoke out against the AstraZeneca vaccine, citing unfounded claims of diminished efficacy in some age groups, a move that curtailed demand for the vaccine and was later said to have contributed to hundreds of thousands of unnecessary deaths in unvaccinated populations (Walsh, 2022).

12. The authors would like to emphasize that the company itself has not confirmed these strategic motivations.

13. Note that contrary to the assumptions of their most vigorous critics, Scherer and colleagues (2013) do acknowledge a role for company self-interest, arguing that to maintain their social license to operate, companies would first try to strategically manipulate public opinion by public relations efforts and by accepting societal standards (e.g., not selling vaccines for unreasonably high prices in this instance). Only when these attempts proved insufficient, would they engage with stakeholders (i.e., COVAX), employing moral reasoning to effect change for societal benefit (i.e., seek more equitable vaccine access).
