# Supplementary material for: Public Health and Political Corporate Social Responsibility: Pharmaceutical Company Engagement in COVAX
Source: Bus Soc. 2023 Apr 13:00076503231158600. doi: 10.1177/00076503231158600 (PMC10102828; doi:10.1177/00076503231158600)
Supplement: sj-docx-1-bas-10.1177_00076503231158600 – Supplemental material for Public Health and Political Corporate Social Responsibility: Pharmaceutical Company Engagement in COVAX [file sj-docx-1-bas-10.1177_00076503231158600.docx]

# Appendix A. Overview of Approved Covid-19 Vaccine Candidates.^1^

| ***Company Name(s)*** | ***Vaccine candidate*** | ***Funding information*** | ***Authorization*** | ***Number of doses distributed in total (as of 31.05.2021)***^2^ | ***Number of doses committed to COVAX*** | ***Access to medicine Index 2021*** |
| --- | --- | --- | --- | --- | --- | --- |
| *Pfizer / BioNTech* | BNT162b2/ COMIRNATY  Tozinameran (INN) | Pfizer did not accept funding from BARDA, BioNTech received $546M US from EU and German government | FDA: 11.12.2021  EMA: 21.12.2020  WHO: 31.12.2021 | 368.68 million doses administered (as of 31.05.2021) | 40 million (agreement on 22.01.2021) | Pfizer: Rank 4; BioNTech: not included |
| *AstraZeneca / University of Oxford* | AZD1222 | AstraZeneca received funding from US government, UK government, CEPI and Gavi (over $1,9Bn US) | EMA: 29.01.2021  WHO: 15.02.2021 | 49 million doses administered (31.05.2021) | Pledged 170 million doses; 550 million through Serum Institute of India (agreement on: 18.12.2020) | Rank 7 |
| *Moderna* | mRNA-1273.351 | Moderna received initial R&D funding from CEPI as well as $2.48Bn from US government | FDA: 18.12.2020  EMA: 06.01.2021  WHO: 30.04.2021 | 149.45 million administered (31.05.2021) | Pledged 500 million doses (agreement on 03.05.2021) | Not included |
| *Janssen (Johnson &Johnson)* | Ad26.COV2.S | Received $1.5Bn from US government | FDA: 27.02.2021  EMA: 11.03.2021  WHO: 12.03.2021 | 14.14 million administered (31.05.2021) | Pledged 500 million doses (agreement on: 18.12.2020) | Rank 3 |
| *Serum Institute of India* | Covishield (ChAdOx1_nCoV-19) | Unknown | WHO: 15.02.2021 | unknown | Pledged 1.1 Bn doses of AstraZeneca or Novavax vaccine (agreement on: 29.09.2020) | Not included |
| *Sinopharm (Beijing Institute of Biological Products Co., Ltd. BIBP)* | SARS-CoV-2 Vaccine (Vero Cell), Inactivated (lnCoV) | Unknown | WHO: 07.05.2021 | 2.83 million administered (31.05.2021) | Negotiations, but no formal agreement (as of 31.05.2021) | Not included |

Notes:

^1^ Source: <https://www.gavi.org/sites/default/files/covid/covax/COVAX%20Supply%20Forecast.pdf> (April 7th 2021 report; accessed 27 May 2021).

^2^ Source: <https://ourworldindata.org/grapher/covid-vaccine-doses-by-manufacturer?tab=table&time=earliest..2021-05-31> (accessed 3 March 2022). All individual administered doses (= vaccine doses injected into people’s arms) are counted, not accounting for vaccination protocols that require multiple vaccinations.

# Appendix B. Timeline of Company Engagement with COVAX.

| **Date** | **Event** |
| --- | --- |
| 24.07.2020 | Access to Covid-19 Tools (ACT) Accelerator launched |
| 30.07.2020 | AstraZeneca and Oxford University sign agreement for the development and distribution of vaccines |
| **05.2020** | **Countries start signing advance purchase agreements with vaccine manufacturers** |
| 21.05.2020 | AstraZeneca mentions engaging with Covax in corporate press release |
| **04.06.2020** | **First Covax AMC: AstraZeneca signs $750m agreement to support the manufacturing, procurement and distribution of 300 million doses of the vaccine** |
| 04.06.2020 | AstraZeneca and SII reach licensing agreement for manufacturing additional vaccine doses for low and middle-income countries |
| 26.06.2020 | Covax mentioned by name for the first time in WHO news section |
| 09.07.2020 | Covax calls for nomination of experts to advise on Covid-19 vaccine candidates |
| 15.07.2020 | 75 countries submit “Expressions of Interest” to engage with Covax as self-financing members |
| **21.09.2020** | **64 high-income countries formally join Covax; funding a gap of around 1.3 billion US$ to secure vaccines for low- and middle-income countries** |
| **29.09.2020** | **SII pledges to produce 100 million AstraZeneca and 100 million Novavax vaccine doses for Covax in 2021** |
| **28.10.2020** | **Sanofi and GlaxoSmithKline pledge 200 million doses of their vaccine to Covax, 300 million doses to the EU and up to 600 million doses to the US** |
| 29.10.2020 | Moderna in talks with Covax to work out a tiered pricing model |
| 09.11.2020 | Positive interim data from Pfizer/BioNTech Phase III clinical trial |
| 16.11.2020 | Positive interim data from Moderna Phase III clinical trial |
| 23.11.2020 | Positive interim data from Oxford/AstraZeneca phase III clinical trial |
| 15.12.2020 | WHO in talks with Pfizer regarding global allocation of vaccines |
| **18.12.2020** | **Covax reaches additional agreements with AstraZeneca and Johnson&Johnson to secure a total of nearly 2 billion vaccine doses.** |
| 06.01.2021 | India issues an export ban for SII’s AstraZeneca shots for several months; intending to vaccinate its own population first |
| 20.012021 | The African Union reaches agreements with SII, AstraZeneca, Pfizer and J&J for a total of 270 million vaccine doses |
| **22.01.2021** | **Covax reaches advance purchase agreement with Pfizer/BioNTech for up to 40 million doses** |
| **03.02.2021** | **SII agrees to provide 1.1 billion doses of AstraZeneca and Novavax vaccines to Covax** |
| 19.02.2021 | Novavax pledges 1.1 billion vaccine doses of its vaccine to Covax |
| **24.02.2021** | **Covax kicks off its global rollout of vaccine doses** |
| **03.05.2021** | **Covax signs advance purchase agreement with Moderna to secure up to 500 million vaccine doses** |
| **06.05.2021** | **Covax reaches advance purchase agreement with Novavax to secure 350 million vaccine doses** |

# Appendix C. Analysed Media Articles.

| **Code** | **Publisher** | **Date** | **Title** |
| --- | --- | --- | --- |
| FP1 | *FiercePharma* | 31.07.2020 | Pfizer, BioNTech keep COVID vaccine deals rolling with 120M-dose Japan pact |
| FP2 | *FiercePharma* | 11.08.2020 | Coronavirus tracker: Sinovac unveils midstage vaccine data; Daewoong OK'd to test tapeworm med against virus |
| FP3 | *FiercePharma* | 26.10.2020 | Uğur Şahin |
| FP4 | *FiercePharma* | 28.10.2020 | Sanofi, GSK to provide 200M coronavirus vaccines to COVAX for equitable distribution |
| FP5 | *FiercePharma* | 30.10.2020 | COVID-19 tracker: Regeneron's antibody cocktail hit by safety concerns; Novo's Rybelsus emerging from pandemic slump |
| FP6 | *FiercePharma* | 12.11.2020 | Generics players join pledge to rapidly scale access to COVID-19 drugs. But will pharma sign on? |
| FP7 | *FiercePharma* | 12.11.2020 | Johnson & Johnson confident in 1B dose goal for COVID-19 vaccine next year, looking ahead to 2022 |
| FP8 | *FiercePharma* | 16.02.2021 | U.K. inspects AstraZeneca vaccine partner's India manufacturing, setting stage for supply boost |
| FP9 | *FiercePharma* | 18.02.2021 | Novavax, coronavirus shot data in hand, strikes an eye-popping supply deal with global vaccine consortium |
| FP10 | *FiercePharma* | 22.02.2021 | AstraZeneca's Indian COVID-19 vaccine partner told to prioritize local supplies: CEO |
| FT1 | *Financial Times* | 06.03.2020 | The $2bn race to find a vaccine |
| FT2 | *Financial Times* | 09.04.2020 | Transcript: Bill Gates speaks to the FT about the global fight against coronavirus |
| FT3 | *Financial Times* | 09.04.2020 | Bill Gates urges rich countries to fund coronavirus vaccine search |
| FT4 | *Financial Times* | 28.04.2020 | Drugmakers race to scale up vaccine capacity |
| FT5 | *Financial Times* | 05.05.2020 | Will poor countries get the coronavirus treatments they need? |
| FT6 | *Financial Times* | 14.05.2020 | Why vaccine ‘nationalism’ could slow coronavirus fight |
| FT7 | *Financial Times* | 29.05.2020 | GSK sets sights on production of 1bn vaccine booster doses |
| FT8 | *Financial Times* | 23.07.2020 | Rich country vaccine rush threatens supply security |
| FT9 | *Financial Times* | 23.07.2020 | Lloyd’s of London to offer Covid-19 vaccine insurance |
| FT10 | *Financial Times* | 19.08.2020 | Rich countries urged to join vaccine facility |
| FT11 | *Financial Times* | 25.08.2020 | Covid vaccine strategy must consider need as well as wealth |
| FT12 | *Financial Times* | 02.09.2020 | ‘Vaccine nationalism’ delays WHO’s struggling Covax scheme |
| FT13 | *Financial Times* | 16.09.2020 | Vaccine fairness will make us all safer |
| FT14 | *Financial Times* | 24.09.2020 | Frontrunners open gap in global race for virus vaccine |
| FT15 | *Financial Times* | 07.10.2020 | FT Health: Fighting vaccine nationalism |
| FT16 | *Financial Times* | 09.10.2020 | Johnson has taken the wrong economic lesson from the crisis |
| FT17 | *Financial Times* | 10.10.2020 | China joins WHO initiative to offer 2bn inoculations |
| FT18 | *Financial Times* | 13.10.2020 | China has broader aims with vaccine diplomacy |
| FT19 | *Financial Times* | 22.10.2020 | Poor countries flex collective muscle to procure drugs |
| FT20 | *Financial Times* | 22.10.2020 | Are we ready for the next pandemic? |
| FT21 | *Financial Times* | 23.10.2020 | How much will a Covid-19 vaccine cost? |
| FT22 | *Financial Times* | 29.10.2020 | GSK on course to hit forecast after standard vaccine boost |
| FT23 | *Financial Times* | 30.10.2020 | Vaccine bond sale raises $500m to fund immunisation programmes |
| FT24 | *Financial Times* | 30.10.2020 | Vaccine bonds: capital idea |
| FT25 | *Financial Times* | 16.11.2020 | The pandemic crisis that’s going to waste |
| FT26 | *Financial Times* | 19.11.2020 | There is a way to keep America globally engaged |
| FT27 | *Financial Times* | 21.11.2020 | Now we need to vaccinate the world |
| FT28 | *Financial Times* | 23.11.2020 | G20 vows to ensure global vaccine supply |
| FT29 | *Financial Times* | 23.11.2020 | How the Oxford-AstraZeneca vaccine works and why it matters |
| FT30 | *Financial Times* | 11.12.2020 | Sanofi/GSK delay vaccine rollout and Australia ditches trial |
| FT31 | *Financial Times* | 14.12.2020 | Patently unfair business of Covid vaccines |
| FT32 | *Financial Times* | 20.12.2020 | Anxiety clouds China’s rush to vaccinate parts of the world |
| FT33 | *Financial Times* | 21.12.2020 | China charm offensive poses threat to confidence in jabs |
| FT34 | *Financial Times* | 22.12.2020 | Opacity dogs Chinese and Russian Covid vaccines |
| FT35 | *Financial Times* | 14.01.2021 | Letter: West’s vaccine rollout must not ignore needs of poorer nations |
| FT36 | *Financial Times* | 21.01.2021 | Unequal vaccine access will return to haunt the rich |
| FT37 | *Financial Times* | 21.01.2021 | Low-income countries trail in global vaccines race |
| FT38 | *Financial Times* | 25.01.2021 | Vaccine delays pose global recovery threat |
| FT39 | *Financial Times* | 26.01.2021 | Davos highlights: European leaders urge Biden to extend efforts to reignite international co-operation |
| FT40 | *Financial Times* | 28.01.2021 | The struggle to defuse the global vaccine conflict |
| FT41 | *Financial Times* | 29.01.2021 | The west’s vaccine myopia |
| FT42 | *Financial Times* | 30.01.2021 | Global co-operation is needed to beat the virus |
| FT43 | *Financial Times* | 02.02.2021 | African countries look beyond west for vaccines |
| FT44 | *Financial Times* | 03.02.2021 | Lessons from the pandemic |
| FT45 | *Financial Times* | 04.02.2021 | Co-operation will help get jabs to the poorest |
| FT46 | *Financial Times* | 06.02.2021 | The global race between vaccines and mutations |
| FT47 | *Financial Times* | 11.02.2021 | Letter: West’s vaccines policies smack of neocolonial hypocrisy |
| FT48 | *Financial Times* | 12.02.2021 | A race to vaccinate the developing world |
| FT49 | *Financial Times* | 12.02.2021 | Iran vaccine drive entangled in geopolitics |
| FT50 | *Financial Times* | 13.02.2021 | Covax challenges exposed as poorer countries fall behind on jabs |
| FT51 | *Financial Times* | 17.02.2021 | The world's biggest test of co-operation |
| FT52 | *Financial Times* | 19.02.2021 | Macron calls for urgent supply of Covid vaccines to poorer nations |
| FT53 | *Financial Times* | 19.02.2021 | G7 leaders vow to boost vaccine supplies to developing world |
| FT54 | *Financial Times* | 20.02.2021 | G7 leaders pledge world vaccine boost |
| FT55 | *Financial Times* | 24.02.2021 | Ghana becomes first country to receive free Covax vaccines |
| FT56 | *Financial Times* | 25.02.2021 | J&J vaccine performs well against variant |
| FT57 | *Financial Times* | 26.02.2021 | African price for Russia vaccine blunts attacks on 'unethical' west |
| F1 | *Forbes* | 01.04.2020 | Coronavirus Business Tracker: How The Private Sector Is Fighting The COVID-19 Pandemic |
| F2 | *Forbes* | 28.10.2020 | GSK And Sanofi Pledge 200 Million Vaccine Doses To Covid-19 Alliance |
| F3 | *Forbes* | 12.11.2020 | Bill And Melinda Gates Are Giving Another $70 Million For Covid Vaccines |
| F4 | *Forbes* | 16.12.2020 | Report: WHO Likely To Fail In Goal To Deliver 2 Billion Coronavirus Vaccine Doses To 92 Lower-Income Countries By End Of 2021 |
| F5 | *Forbes* | 15.02.2021 | AstraZeneca/Oxford Covid-19 Vaccine Gets Emergency Approval From WHO |
| F6 | *Forbes* | 18.02.2021 | Biden To Offer $4 Billion To Fund Coronavirus Vaccinations In Developing Countries |
| F7 | *Forbes* | 24.02.2021 | Ghana Becomes First Nation To Receive Vaccines From WHO-Backed Covax Initiative |
| F8 | *Forbes* | 25.02.2021 | Yellen Urges World’s Largest Economies To ‘Go Big’ On Stimulus |
| PT1 | *PharmaTimes* | 05.06.2020 | AZ ramps up supply capacity for COVID-19 vaccine |
| PT2 | *PharmaTimes* | Oct 2020 | Pandemic priorities (published in Oct 2020 Magazine) |
| PT3 | *PharmaTimes* | 28.10.2020 | Sanofi and GSK to provide COVID-19 vaccine doses to COVAX Facility |
| PT4 | *PharmaTimes* | Jan/Feb 2021 | Expectations for healthcare in 2021 (published in Jan/Feb 2021 Magazine) |
| ET1 | *The Economic Times* | 05.06.2020 | AstraZeneca & Serum Institute of India sign licensing deal for 1 billion doses of Oxford vaccine |
| ET2 | *The Economic Times* | 16.07.2020 | India Must Push for Fair Share of Covid Vaccines' |
| ET3 | *The Economic Times* | 24.07.2020 | India's keen to join WHO- Gavi vaccine alliance in bid to keep supply cost effective |
| ET4 | *The Economic Times* | 04.08.2020 | GAVI in talks to make Indian vaccine makers join Covax: CEO Seth Berkley |
| ET5 | *The Economic Times* | 05.08.2020 | Lack of Norms Makes Vaccine-making Hard' |
| ET6 | *The Economic Times* | 07.08.2020 | Globally, Cos can Produce 2-4 b Covid Vaccine Doses by 2021 End' |
| ET7 | *The Economic Times* | 17.09.2020 | India to be a big hub for production of Russian vaccines: Kirill Dimitriev, CEO, RDIF |
| ET8 | *The Economic Times* | 03.11.2020 | India now has Rights for Vaccines to Cover its 50% of Population' |
| ET9 | *The Economic Times* | 11.11.2020 | Pfizer's coronavirus vaccine isn't expected to reach India anytime soon; US, UK first in line |
| ET10 | *The Economic Times* | 13.11.2020 | Pfizer starts talks with Covax facility for equitable distribution of COVID-19 vaccine |
| ET11 | *The Economic Times* | 16.11.2020 | Vaccine hoarding could prolong Covid-19 till 2024 |
| ET12 | *The Economic Times* | 18.11.2020 | India's likely to get access to Moderna's coronavirus vaccine via COVAX facility |
| ET13 | *The Economic Times* | 19.12.2020 | IFC invests $30m in Indian vaccine maker Biological E for future Covid vaccines, routine immunisation |
| ET14 | *The Economic Times* | 30.12.2020 | 40-50 million Covid vaccine stockpiled by SII, India to get most of it |
| ET15 | *The Economic Times* | 06.01.2021 | No Ban on Export of Covid-19 Vaccines' |
| ET16 | *The Economic Times* | 06.01.2021 | No Decision Yet on Indemnity Waiver to Vaccine Cos |
| ET17 | *The Economic Times* | 15.01.2021 | Covid vaccine makers be liable for adverse effects of jabs |
| ET18 | *The Economic Times* | 20.01.2021 | Covid: WHO criticises countries, vaccine companies over bilateral deals |
| ET19 | *The Economic Times* | 20.01.2021 | India cites global shortage of Covid-19 vaccines as consensus eludes its TRIPS waiver proposal |
| ET20 | *The Economic Times* | 23.01.2021 | View: India’s vaccine internationalism is in perfect alignment with its role as a major global player |
| ET21 | *The Economic Times* | 26.01.2021 | Tata in talks to launch Moderna vaccine in India |
| ET22 | *The Economic Times* | 02.02.2021 | India-made Covishield part of Pakistan jab drive under vaccine alliance |
| ET23 | *The Economic Times* | 17.02.2021 | Vaccine diplomacy: 37% doses exported by India are grants |
| ET24 | *The Economic Times* | 17.02.2021 | South Africa asks SII to take back 1 mn Covid vaccines |
| WSJ1 | *The Wall Street Journal* | 04.06.2020 | AstraZeneca Signs More Coronavirus Vaccine Supply Deals; Under one of the deals, drug maker will receive $750 million from two foundations to supply 300 million doses for global distribution |
| WSJ2 | *The Wall Street Journal* | 02.09.2020 | Rich Nations Snap Up First Vaccine Doses |
| WSJ3 | *The Wall Street Journal* | 25.11.2020 | New Covid-19 Vaccines Stir Hope for the World's Poor; Promising trial results inspire optimism in the developing world, which could yet become a reservoir for the virus for years to come |
| WSJ4 | *The Wall Street Journal* | 24.12.2020 | Who Made the Vaccine Possible? Not WHO |
| WSJ5 | *The Wall Street Journal* | 07.01.2021 | South Africa Fast Tracks Regulatory Approval to Secure Covid-19 Vaccines; Low- and middle-income countries are expected to emulate the move to expedite getting shots to at least some at-risk citizens |
| WSJ6 | *The Wall Street Journal* | 07.01.2021 | Israel Races Ahead With Covid-19 Vaccines, While Palestinians Wait; The differing pictures raise questions for Israel about its obligations to the West Bank and Gaza |
| WSJ7 | *The Wall Street Journal* | 04.02.2021 | Tanzania Rejects Shot Against 'Satanic' Virus |
| WSJ8 | *The Wall Street Journal* | 15.02.2021 | WHO Approves AstraZeneca Covid-19 Vaccine for Emergency Use; Listing clears way for delivery of free shots to developing nations |
| WSJ9 | *The Wall Street Journal* | 16.02.2021 | World News: WHO Approves AstraZeneca Shot For Emergency Use |
| WSJ10 | *The Wall Street Journal* | 18.02.2021 | World News: Delayed Developing-Nation Shots Risk Extending Crisis |
| WSJ11 | *The Wall Street Journal* | 25.02.2021 | World News: Ghana First to Get Doses Under WHO Plan |
